# Topical MTII Therapy Suppresses Melanoma Through PTEN Upregulation and Cyclooxygenase II Inhibition

**DOI:** 10.3390/ijms21020681

**Published:** 2020-01-20

**Authors:** Jian-Ching Wu, Han-En Tsai, Yi-Hsiang Hsiao, Ji-Syuan Wu, Chieh-Shan Wu, Ming-Hong Tai

**Affiliations:** 1 Biobank and Tissue Bank, Kaohsiung Chang Gung Memorial Hospital, Kaohsiung 83301, Taiwan; djbluestyle338@hotmail.com; 2 Doctoral Degree Program in Marine Biotechnology, National Sun Yat-sen University, 70 Lien-Hai Road, Kaohsiung 80424, Taiwan; 3 Doctoral Degree Program in Marine Biotechnology, Academia Sinica, 128 Academia Road, Section 2, Nankang, Taipei 11529, Taiwan; 4 Institute of Biomedical Science, National Sun Yat-sen University, Kaohsiung 80424, Taiwan; racheltsai0713@gmail.com (H.-E.T.); black156743@gmail.com (Y.-H.H.); sunny18130215@gmail.com (J.-S.W.); 5 Department of Dermatology, Kaohsiung Veterans General Hospital, Kaohsiung 81362, Taiwan; 6 Department of Dermatology, Faculty of Medicine, College of Medicine, Kaohsiung Medical University, Kaohsiung 81362, Taiwan; 7 Center for Neuroscience, National Sun Yat-sen University, Kaohsiung 80424, Taiwan

**Keywords:** MTII, Melanoma, PTEN, COX-2, PGE2

## Abstract

Melanotan II (MTII), a synthetic analogue of the alpha-melanocyte stimulating hormone (α-MSH), has been applied for skin tanning in humans. However, the carcinogenic consequence of topical MTII has been equivocal. This study aims to delineate the anti-neoplastic efficacy and mechanism of MTII using the B16-F10 melanoma model in vitro and in vivo. It was found that, despite a lack of influence on proliferation, MTII potently inhibited the migration, invasion, and colony-forming capability of melanoma cells. Moreover, topical MTII application significantly attenuated the tumor progression in mice bearing established melanoma. Histological analysis revealed that MTII therapy induced apoptosis while inhibiting the proliferation and neovaluarization in melanoma tissues. By immunoblot and immunohistochemical analysis, it was found that MTII dose-dependently increased the phosphatase and tensin homolog (PTEN) protein level while reducing PTEN phosphorylation, which resulted in the inhibition of AKT/nuclear factor kappa B (NFκB) signaling. Consistently, MTII treatment inhibited cyclooxygenase II (COX-2) expression and prostaglandin E2 (PGE2) production in melanoma cells. Finally, studies of antibody neutralization suggest that the melanocortin 1 receptor (MC1R) plays a critical role in MTII-induced PTEN upregulation and melanoma suppression. Together, these results indicate that MTII elicits PTEN upregulation via MC1R, thereby suppressing melanoma progression through downregulating COX-2/PGE2 signaling. Hence, topical MTII therapy may facilitate a novel therapeutic strategy against melanoma.

## 1. Introduction

Melanoma is a frequently diagnosed skin cancer, with an estimated 91,270 new cases in the United States in 2018 [1]. Early stage melanoma is usually curable by surgical resection and radiotherapy, with a five-year survival rate around 90%. However, once developed into malignant melanoma, the prognosis becomes poor, so that the five-year survival rate drops to 17%. [2]. Melanoma development is frequently accompanied with several genetic alternations, including *N-RAS* and *BRAF* mutation, *PTEN*, *CDKN2A,* and *E-cadherin* loss [3]. However, different from in Europe and the United States, acral melanoma is often diagnosed in Asia with distinct genetic alterations, probably due to the difference in genetic background and lifestyle [4,5,6]. Therefore, apart from the current focus on receptor tyrosine kinases (RTKs) and mutant BRAF^V600E^, other therapeutic strategies are needed for melanoma control.

The constitutive activation of phosphoinositide-3-kinase (PI3K)/AKT kinase signaling cascade is one of the prominent routes that contribute to melanoma development. It has been shown that the PI3K/AKT axis modulates several important downstream signaling pathways and transcriptional factors, including the mammalian target of rapamycin (mTOR), nuclear factor-kappaB (NFκB), and hypoxia-inducible factor 1-alpha (HIF-1α), which ultimately stimulates angiogenesis and cell proliferation as well as anti-apoptosis [7]. Among the PI3K/AKT axis, phosphatase and tensin homolog (PTEN) plays a key catalytic role in the conversion of the phosphatidylinositol 3,4,5-trisphosphate (PIP_3_) to PIP_2_, by which negatively regulates AKT signaling activation [8]. The catalytic activity of PTEN protein is dependent on phosphorylation at the PTEN C2 domain, including Ser-380, Thr-382, Thr-383, and Ser-385 [9,10], whereas PTEN stability is controlled by ubiquitination at residues Lys-13, Lys-27, Lys-66, and Lys-289 [11,12,13]. Recent studies indicate that PTEN not only regulates DNA repair through the AKT/p38 signaling axis in the cytoplasm [14] but also translocates into the nucleus to confer chromosome stability against DNA damage by modulating p53 activity and inhibiting nuclear AKT activation [15,16]. In melanocytes during UV exposure, PTEN is upregulated by alpha-melanocyte stimulating hormone (α-MSH)/melanocortin-1 receptor (MC1R) signaling, thereby reducing oxidative stress and DNA damage [17].

Cyclooxygenases (COXs) are a family of myeloperoxidases located at the luminal side of the endoplasmic reticulum and nuclear membrane. The COX family catalyzes the rate-limiting step in the conversion of arachidonic acid to prostaglandins and it has been identified two isoforms: COX1 is constitutively expressed in mammalian tissues and cells, whereas COX-2 is rarely expressed in most normal tissues but is highly inducible in response to various stimuli, such as inflammation reactions. COX-2 is frequently expressed in various tumors including malignant melanomas and its level is correlated with poor prognosis and tumor progression [18,19]. Prostaglandin E2 (PGE2) is one of the major products of COX-2, which is known to modulate cell proliferation, apoptosis, and cell motility in many types of tumors [20]. Cumulative evidences reveal that COX-2 inhibition is efficient to elicit the inhibition of proliferation, migration, and invasiveness of melanoma cells [21,22,23]. Consistently, our previous studies have demonstrated that the gene delivery of proopiomelanocortin (POMC), the precursor of alpha-melanocyte stimulating hormone (α-MSH), effectively suppresses the progression and metastasis of melanoma though the inhibition of COX-2/PGE2 signaling [24]. Among the POMC-derived peptides, α-MSH has been delineated to participate in the anti-neoplastic mechanism of POMC gene therapy via inflammation inhibition [24], neovascularization blockade [25,26], and sensitizing cancer cells to hypoxia-induced apoptosis [27].

Melanotan II (MTII) is a synthetic cyclic heptapeptide analogue of anti-inflammatory peptide α-MSH. As a sun-tanning agent and non-selective agonist of melanocortin receptors (MCRs), MTII is more potent and stable than the endogenous α-MSH [28]. Interestingly, MTII has been proposed to hold therapeutic potential for erectile dysfunction and female arousal and orgasmic disorders [29]. However, the safety and oncogenic potential of MTII remain equivocal [30]. Therefore, the present study evaluates the effects of MTII on the oncogenic behaviors of the B16-F10 melanoma cell in vitro. Subsequently, we investigate whether topical MTII application halts the progression of established B16-F10 melanoma in mice.

## 2. Materials and Methods

### 2.1. Cell Cultures and Reagents

Mouse (B16-F10) and human (A375 and A2058) melanoma cells were purchased from American Type Culture Collection (Manassas, VA, USA). These cells were cultured in DMEM (Invitrogen; Carlsbad, CA, USA) medium containing 10% fetal bovine serum (FBS; Hyclone, Logan, UT, USA), 2 mM glutamine, 100 mg/mL streptomycin (Invitrogen; Carlsbad, CA, USA) and 100 U/mL penicillin at 37 °C in 5% CO_2_ atmosphere. The following antibodies were purchased from Santa Cruz Biotechnology (Santa Cruz, CA, USA): p-PTEN (sc-377573), PTEN (sc-7974), p-AKT (sc-293125), AKT (sc-5298), COX-2 (sc-70879), NFκB p65 (sc8008), MC1R (sc-28990). Antibody against β-actin (A5441) was purchased from Sigma–Aldrich (St. Louis, MO, USA). The antibody against Ki67 (MA5-14520) was purchased from Thermo Fisher Scientific (Waltham, MA, USA). The antibody against CD31 (NCL-L-CD31-607) was purchased from Leica Biosystems (Newcastle Upon Tyne, UK). The MTII peptide was purchased from Kelowna International Scientific Inc. (San Chung District, New Taipei City, Taiwan).

### 2.2. Cell Proliferation Assay

Cells were cultured in a 96-well plate at a density of 1 × 10^4^ cells/mL. After treatment with MTII peptides at the indicated concentration for 48 h, cells were incubated with 3-(4,5-dimethylthiazol-2-yl)-2,5-diphenyltetrazolium bromide (MTT; 0.5 mg/mL) for 2 h at 37 °C. The formazan in viable cells was dissolved with 100 μL of dimethyl sulfoxide and determined by reading optical densities in a microplate reader (Dynex Technologies, Inc., Chantilly, VA, USA) at an absorption wavelength of 570 nm.

### 2.3. Colony Formation Assay

B16-F10 cells in 6-well plates (1 × 10^3^ cells per well) were incubated with the indicated concentration of peptides once every two days in 5% fetal calf serum medium for 7 days. For antibody neutralization experiments, cells were simultaneously treated with MTII (10 nM) and MC1R antibody (2 μg/mL) once every two days in fresh serum medium for one week. After treatment with MTII, cells were fixed in 4% paraformaldehyde and incubated in crystal violet (0.01% in 10% buffered formalin; Sigma–Aldrich, St. Louis, MO, USA) for 30 min for colony counting.

### 2.4. Cell Invasion Assay

Cell invasion assay was assessed using a Boyden chamber and was performed as previously described [31]. Briefly, a polycarbonate filter (8 μm pore size Nucleopore; Costar, Cambridge, MA, USA) was coated with Matrigel (1:2 dilution; BD Biosciences, San Jose, CA, USA) in advance, and then B16-F10 melanoma cells were counted at 3 × 10^5^ cells/mL in serum free DMEM medium containing indicated concentration of peptides. After treatment, the cell mixtures were loaded 50 μL into the upper chamber, while the lower chamber was supplemented with complete medium. The Boyden chamber was placed in a humidified CO_2_ incubator at 37 °C for 16 h. Traversed cells were fixed by methanol for 10 min and stained with 10% Giemsa solution (Sigma–Aldrich, St. Louis, MO, USA) for 20 min. The cell number was counted from three different low power fields and expressed as the mean ± SD.

### 2.5. Scratch Migration Assay

The migration ability of B16-F10 melanoma cells were assessed using a scratch wound healing assay. Briefly, a gap of approximately 1 mm was created in the adherent layer of confluent B16-F10 cells (in six-well plates) by a sterile 0.1 mL pipette tip (Gilson, Inc., Middleton, WI, USA). Next, cells were treated with α-MSH and MTII peptides at a concentration of 10^−8^ M. The closure extent of the cell-free gap was recorded at 2 h intervals for 14 h by microscopy.

### 2.6. Primary Melanoma Model

All animal experiments were carried out under protocols approved by the Animal Care and Use Committee (IACUC) of National Sun Yet-Sen University. To induce the primary melanoma, B16-F10 cells were subcutaneously inoculated into the back of C57BL/6 mice (5 × 10^5^ cells in 0.1 mL of medium). After implantation for 7 days, tumor-bearing mice were randomly divided into control and MTII groups (*n* = 6). Each melanoma in mice was treated daily with a topical MTII peptide (0.1 mL of 1 μg/mL MTII in PBS) for 10 days. The tumor volumes were measured with a dial-caliper according to the following formula: width^2^ × length × 0.52.

### 2.7. Immunohistochemistry Analysis

The resected melanoma tissues were analyzed according to the proliferation index, PTEN, NFκB, and COX-2 expressions by Ki67 and indicated antibodies, respectively. Briefly, after deparaffinization, the resected tissues were blocked with 3% hydrogen peroxide for 10 min and retrieved the antigen epitopes by microwave in 10 mM citrate buffer for 30 min. The indicated antibodies were applied onto the sections and incubated at 4 °C overnight followed by repeated washing with PBS. Horseradish peroxidase/Fab polymer conjugate (Polymer detection system; Invitrogen/Zymed, Carlsbad, CA, USA) was applied to the sections and the sections were incubated for 30 min. Finally, the sections were incubated with peroxidase substrate diaminobenzidine (1:20 dilution, Zymed) and counterstained with Gill’s hematoxylin before dehydration.

### 2.8. Immunofluorescence Staining of Fixed Tumor Sections

After deparaffinization and antigen retrieval, the slides were blocked with solution containing 5% BSA (*v/v*) and 0.1% Triton X100 (*v/v*) for 30 min and incubated with CD31 antibody at 4 °C overnight. After washing with PBS, the sections were incubated with Alexa Fluor-conjugated secondary antibody (Invitrogen; Carlsbad, CA, USA) for 1 h and subsequently counterstained with DAPI for 10 min and viewed under a fluorescent microscope.

### 2.9. Terminal Deoxynucleotidyl Transferase (TdT) Mediated dUTP Nick End Labeling (TUNEL) Assay

Cell death in paraffin-embedded mouse melanoma sections was detected by enzyme labelling of DNA strand breaks using a TUNEL assay (In Situ Cell Death Detection Kit; Roche, Mannheim, Germany) according to the manufacturer’s instructions. Briefly, sections were incubated with a mixture of terminal transferase (TdT) and fluorescein-dUTP reaction at 37 °C to label free 3′OH ends of genomic DNA. After TUNEL staining, sections were counterstained with 4′,6-diamidino-2-phenylindole, dihydrochloride (DAPI) for 20 min and viewed under a fluorescent microscope. For quantifying the immunostaining of TUNEL, each sample was randomly captured by microscopy for three independent fields. The apoptotic percentage was estimated by normalizing the number of TUNEL-positive cells to the total number of DAPI-positive cells and calculated from three independent fields with a 100× microscopic field.

### 2.10. NFκB Luciferase Assay

B16-F10 cells were co-transfected with the NFκB-driven luciferase (Stratagene, La Jolla, CA, USA) vector and the *Renilla reniformis* luciferase reporter vector (Promega, Madison, WI, USA) at a ratio of 1:1/10 using Lipofectamine 3000 Reagent (Invitrogen; Carlsbad, CA, USA) for 4 h before being maintained with fresh medium for 24 h. Subsequently, cells were treated with MTII (10 nM) with or without lipopolysaccharide (LPS; 100 ng/mL) for 24 h. The NFκB-driven luciferase activities in cells were measured using a dual light kit (Promega, Madison, WI, USA) in Orion II microplate luminometer (Titertek Berthold; Pforzheim, Germany) and normalized with that of *R. reniformis* luciferase according to the manufacturer’s instructions.

### 2.11. Secreted PGE2 Measurement

PGE2 concentrations in cell culture supernatants were determined by commercial ELISA kit (Cayman Chemical Company, Ann Arbor, MI, USA) according to the manufacturer’s instructions. Briefly, after supernatant collection, the total cellular proteins were extracted and then measured by bicinchoninic acid (BCA) assay to assess the different cell numbers of the different groups. The secreted PGE2 levels were normalized to the total cellular protein and expressed as the mean ± SD.

### 2.12. Western Blot Analysis

Cell lysates were prepared and the level of protein expression was measured as previously described [32]. The PVD membrane was blocked with 5% milk in TBS-T for 1 h then incubated with specific primary antibodies and secondary antibodies conjugated with HRP (1:10,000 dilutions in 5% milk) for 1 h respectively. The signals on the membrane were detected using chemiluminescent HRP substrate (Millipore Corporation; Billerica, MA, USA) and exposed to X-ray film for the autoradiogram.

### 2.13. Quantitative Real-Time PCR

RNA was purified and quantitative real-time PCR was performed as previously described [32]. The cDNA product was used for quantitative real-time PCR by the SYBR Green PCR master mix and the predesigned gene-specific probe and primer sets for mouse COX-2 (NM_011198.4). Data were normalized to β-actin (NM_007393.3) and expressed as fold changes. The primer sequences were as follows: COX-2 forward primer (5′-GGT GTA TCC CCC CAC AGT CA-3′) and reverse primer (5′-CCA GGC ACC AGA CCA AAG AC-3′); β-actin forward primer (5′-GGA ATC CTG TGG CAT CCA T-3′) and reverse primer (5′-GCT CAG GAG GAG CAA TGA T-3′).

### 2.14. Statistical Analysis

All data are expressed as the mean ± standard deviation (SD). Statistical analysis was performed with one-way ANOVA followed by Newman–Keuls post hoc or t-test (for multiple comparisons) using Prism version 5 (GraphPad Software, Inc., La Jolla, CA, USA). A *p* value of less than 0.05 was considered statistically significant.

## 3. Results

### 3.1. MTII Potently Suppressed the Invasiveness and Colony-Forming Capabilities of B16-F10 Melanoma Cells

**Figure 1:**
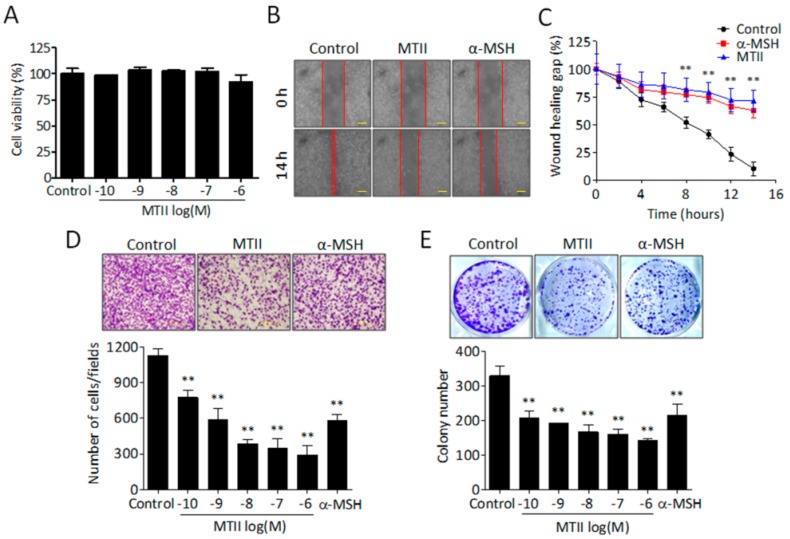
Effect of MTII on tumorigenicity of melanoma cells. (**A**) Cell viability was determined by MTT assay. Data were presented as the mean ± SD from triplicate experiments. (**B**,**C**) After cells were treated with MTII and α-MSH peptides at the concentration 10 nM, the cell migration capability was determined by scratch wound healing assay. The average of the gap width was measured from different duplication experiments and expressed as the mean ± SD. (**D**) After cells were treated with MTII (0.1 nM–1 μM) and α-MSH peptides (10 nM), respectively, the cell invasive ability was determined by Boyden chamber using a Matrigel-coated membrane. The number of invaded cells was calculated from three different fields for each experimental group and expressed as the mean ± SD. (**E**) Cells treated with the indicated concentration of MTII and α-MSH peptides (10 nM), respectively, for 7 days. Cell colonies were visualized by crystal violet staining. **: *p* < 0.01.

To investigate the anti-neoplastic potential of MTII, we evaluated the effect of MTII on the proliferation, migration, invasion, and anchorage-independent growth of B16-F10 melanoma cells. Despite the lack of effect on proliferation (Figure 1A), MTII significantly attenuated the migration (Figure 1B,C) and dose-dependently inhibited the invasiveness (Figure 1D) of B16-F10 melanoma cells. Moreover, MTII potently and dose-dependently suppressed the anchorage-independent growth of melanoma cells at as low as 0.1 nM (Figure 1E). These results suggest that MTII inhibited the oncogenic behaviors of B16-F10 melanoma cells with marginal influence on viability.

### 3.2. Topical MTII Application Attenuated the Progression of Established Melanoma in Mice

**Figure 2:**
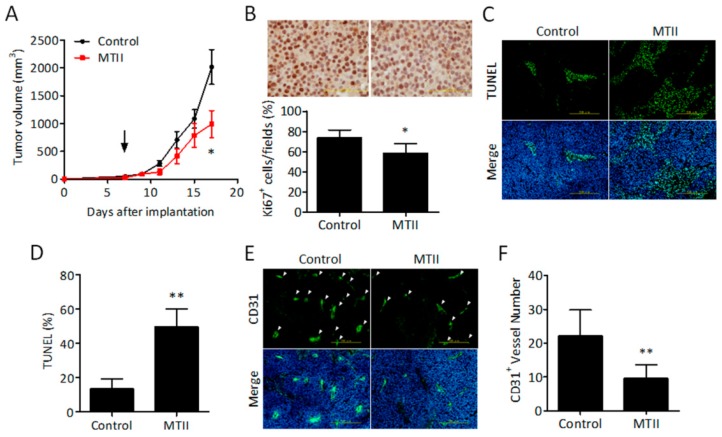
Effect of topical MTII administration on tumor growth in established primary melanoma model. (**A**) Mice were implanted with B16-F10 melanoma cells at Day 0. After the tumor size in each mouse was at least 100 mm^3^ at Day 10, mice were randomly divided into two groups (*n* = 6) and treated with MTII peptide topically once daily for 14 days. The tumor volume was measured by caliper once every two days and presented as the mean ± SEM. The arrow indicates the date of topical MTII treatment. (**B**) The representative images of Ki67 expression in the control and MTII-treated melanoma tissues. The bar chart shows the proliferation index of Ki67 nucleus positive cells in tumor tissues (*n* = 6/each group). Scale bar, 200 μm. (**C**) Representative profiles of TUNEL staining in control and MTII-treated melanoma tissues. DAPI (blue) was used as a nuclear counterstain. Scale bar, 500 μm. (**D**) The apoptotic cells in melanoma tissues are expressed as a percentage of the number of TUNEL positive cells over the number of TUNEL positive cells. Data are expressed as the mean ± SD from 6 experiments. (**E**) Representative profiles of CD31 staining in control and MTII-treated melanoma tissues. Scale bar, 500 μm. (**F**) The amounts of CD31^+^ vessels were counted from different melanoma tissues (*n* = 6). *: *p* < 0.05, **: *p* < 0.01.

We then evaluated the effect of topical MTII application on the growth of established B16-F10 melanoma in mice. It was found that a 10-day MTII therapy significantly perturbed the progression of melanoma in mice, so that the tumor size of MTII-treated melanoma was about 50% of the control group (2016.8 vs. 989.19 mm^3^; Figure 2A). By immunostaining analysis of proliferation index Ki-67, it was found that the Ki-67-positive cells was significantly decreased in MTII-treated melanoma compared with the control (Figure 2B). This was accompanied with a significant increase in TUNEL-positive apoptotic cells (Figure 2C,D) and a reduction in the number of CD31-positive neovascularized microvessels (Figure 2E,F) in MTII-treated melanoma tissues. Together, these findings indicate that topical MTII therapy effectively suppresses, but does not stimulate, the progression of melanoma in mice through proliferation inhibition, apoptosis induction, and neovascularization blockade.

### 3.3. MTII Augmented the PTEN Expression and Repressed the Akt/NFκB Signaling in B16-F10 Melanoma Cells

**Figure 3:**
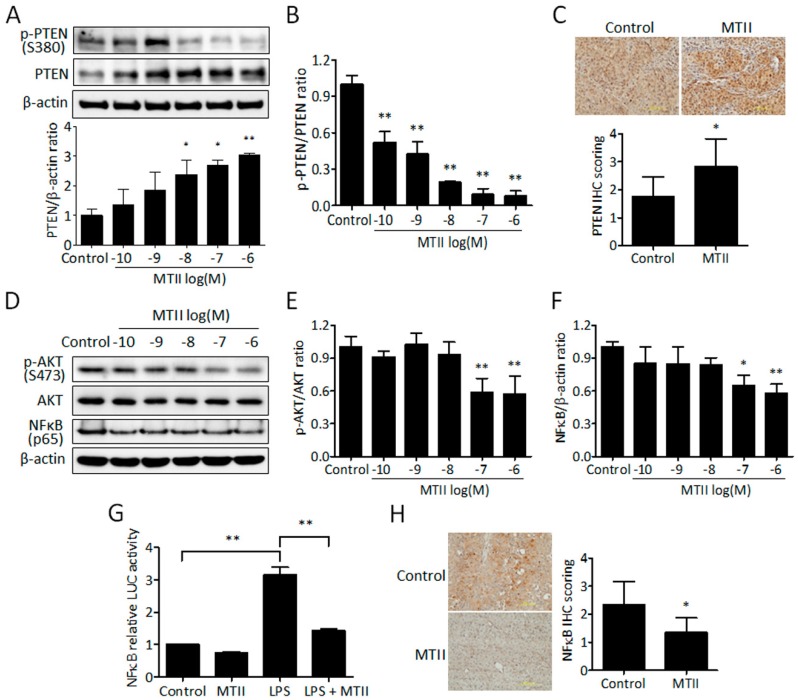
Effect of MTII peptide on the PTEN/AKT/NFκB signaling pathway in melanoma. (A) After being incubated with MTII peptide for 24 h, cells were harvested and analyzed by Western blot analysis. The protein ratio of PTEN was normalized with β-actin by Image Pro-plus analysis software. (**B**) The p-PTEN/PTEN ratio was calculated using Image Pro-plus analysis software from duplicate experiments. The protein ratio of PTEN was normalized with β-actin by Image Pro-plus analysis software. Data was expressed as fold change ± SD of duplicate experiments. (**C**) IHC analysis of PTEN expression in control and MTII-treated melanoma tissues. Scale bar, 200 μm. Data were calculated from different melanoma tissues and expressed as mean ± SD (*n* = 6). (**D**) Western blot analysis of p-AKT, AKT and NFκB (p65) in MTII-treated cells. β-actin was used for normalized protein level. (**E**) The p-AKT/AKT ratio was calculated using Image Pro-plus analysis software from duplicate experiments. (**F**) The protein ratio of NFκB (p65) was normalized with β-actin by Image Pro-plus analysis software. Data were expressed as the fold change ± SD of duplicate experiments. (**G**) Cells were transfected with plasmids encoding a NFκB luciferase report gene for 24 h and then treated with MTII peptide with or without LPS for another 24 h. NFκB activities were measured and expressed as means ± SD from triplicate experiments. (**H**) IHC score analysis of NFκB p65 expression in control and MTII-treated melanoma tissues (*n* = 6). Scale bar, 200 μm. *: *p* < 0.05, **: *p* < 0.01.

Because α-MSH has been shown to stimulate PTEN signaling via MC1R in melanocytes [17], we delineated whether PTEN upregulation participated in the mechanism underlying MTII-mediated melanoma suppression. Immunoblot blot analysis revealed that MTII potently and dose-dependently increased the PTEN protein levels (Figure 3A) but decreased the expression level of inactive, phosphorylated PTEN (pPTEN; Figure 3B) in B16-F10 melanoma cells. Consistently, a higher PTEN immunostaining was observed in MTII-treated B16-F10 melanoma tissues compared with the control (Figure 3C).

Subsequently, we assessed the effect of MTII on the expression profile of downstream effectors of PTEN, including Akt and nuclear factor kappa B (NFκB) in melanoma cells. Immunoblot analysis showed that MTII significantly inhibited the Akt phosphorylation and NFκB p65 expression (Figure 3D–F). The later finding was further validated using NFκB-driven luciferase assay, in which MTII significantly diminished the basal and LPS-induced NFκB activation in melanoma cells (Figure 3E). Histological analysis also revealed a reduced NFκB p65 expression in MTII-treated melanoma tissues compared with control (Figure 3F). These data strongly support that MTII enhances PTEN expression through MC1R and inhibits Akt/NFκB signaling in melanoma cells.

### 3.4. MTII Inhibited COX-2 Expression and PGE2 Production in Melanoma Cells

**Figure 4:**
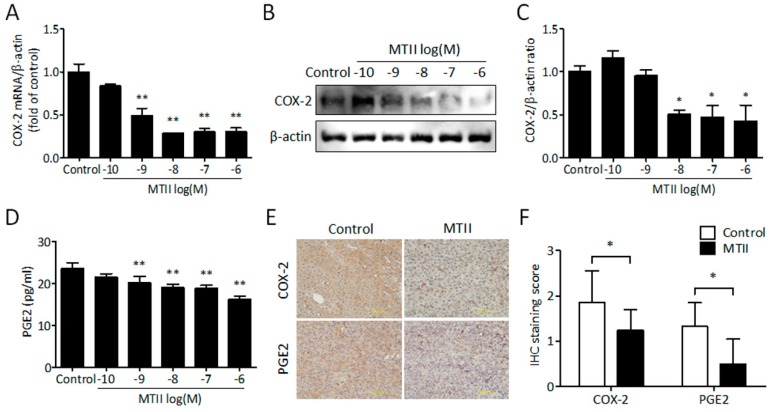
Effect of MTII peptide on COX-2 expression and PGE2 production in melanoma. (**A**) Relative COX-2 mRNA expressions were analyzed by real-time PCR. Data were expressed as means ± SD of the fold change compared with the control from duplicate experiments. (**B**) Western blot analysis of COX-2 expression in MTII-treated B16-F10 melanoma cells. (**C**) The bar chart shows the protein ratio from duplicate experiments. The data are expressed as fold changes ± SD. (**D**) The levels of secreted PGE2 protein (pg/mL) were determined by ELISA assay kit from triplicate experiments. (**E**) Representative profiles of COX-2 and PGE2 expression in control and MTII-treated melanoma tissues. Scale bar, 200 μm. (**F**) The bar char was calculated and expressed as the mean ± SD from six experiments. *: *p* < 0.05, **: *p* < 0.01.

Since the inhibition of NFκB/COX-2 signaling is involved in POMC-mediated melanoma suppression [24], we elucidated whether MTII also perturbed COX-2 expression as well as prostaglandin E2 (PGE2) secretion in B16-F10 melanoma cells. By Western blot analysis, it was found that MTII potently and dose-dependently reduced the COX-2 expression at mRNA (Figure 4A) and protein levels (Figure 4B,C) in melanoma cells. Accordingly, the secreted levels of PGE2 were diminished following exposure to the MTII peptide in the condition medium of B16-F10 melanoma cells (Figure 4D). This inhibitory effect was also observed in MTII-treated melanoma tissues compared with the control group (Figure 4E,F). These data demonstrate that MTII application attenuates the COX-2/PGE2 signaling in melanoma cells.

### 3.5. Antibody Neutralization of MC1R Abolished the MTII-Induced Melanoma Suppression

**Figure 5:**
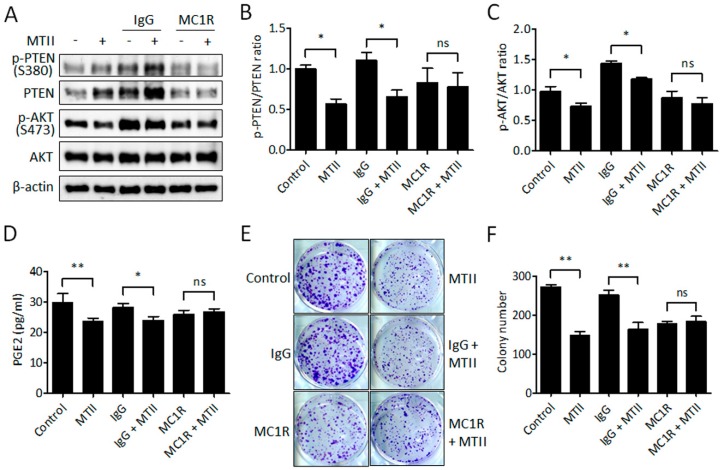
Effect of MC1R antibody neutralization on MTII-induced inhibition of PTEN phosphorylation and colonies formation. (A) Western blot analysis of PTEN and AKT expressions in cells incubation with the MTII peptide and MC1R antibody. β-actin was used for the normalized protein level. (**B**,**C**) The bar chart shows the protein ratio from duplicate experiments. The data are expressed as fold changes ± SD. (**D**) The levels of secreted PGE2 protein (pg/mL) were detected by ELISA assay kit from triplicate experiments. (**E**) Representative images of crystal violet staining in cells received MTII peptide and MC1R antibody challenge compared with control group. (**F**) The amounts of cell colonies were calculated from different triplication experiments and expressed as mean ± SD. *: *p* < 0.05, **: *p* < 0.01; ns, no significance

To investigate whether MTII-induced melanoma suppression was through the MC1R-mediated signaling pathway, we exploited antibody neutralization to block the interaction between MTII and MC1R in B16-F10 melanoma cells. Western blot analysis showed that MC1R antibody neutralization significantly abrogated the MTII-induced reduction in PTEN/AKT phosphorylation (Figure 5A–C). Similarly, the decreased PGE2 production by MTII was restored by MC1R antibody (Figure 5D). Finally, by colony formation assay, it was revealed that MC1R antibody neutralization attenuated the MTII-mediated inhibition of anchorage-independent growth in melanoma cells (Figure 5E,F). Together, these data results indicate that MC1R participates in the mechanism underlying MTII-mediated PTEN upregulation and AKT/COX-2 inhibition.

## 4. Discussion

**Figure 6:**
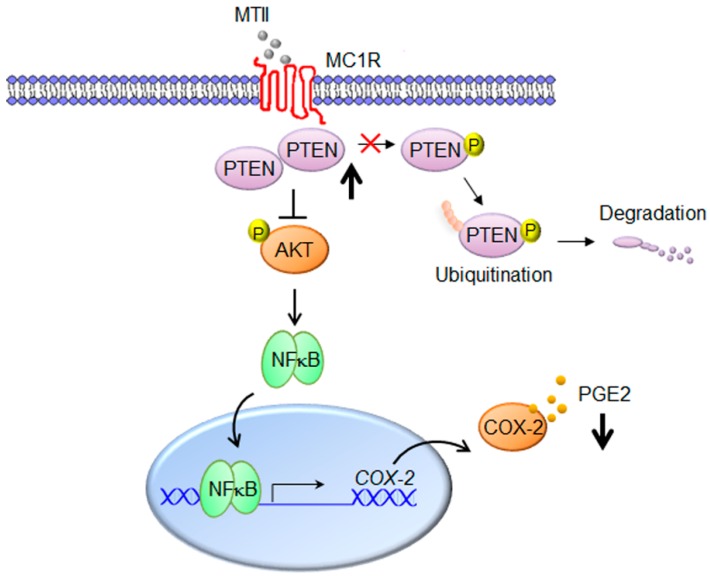
The working model for MTII-mediated melanoma suppression via PTEN upregulation and COX-2/PGE2 inhibition. After binding to MC1R, MTII elevates the cellular PTEN levels by blunting the PTEN degradation pathways, such as phosphorylation and ubiquitination. Subsequently, the escalated PTEN expression perturbs the AKT activation and NFκB-mediated COX-2 expression and PGE2 release.

Despite the advancement in medical technologies, malignant melanoma remains one of the most refractory cancers today. The present study provides first evidence that topical application of the sun-tanning agent MTII suppresses, rather than stimulates, the progression of established melanoma in a preclinical melanoma model. Moreover, MTII administration elicited the anti-melanoma function through the multiple cellular mechanism including proliferation inhibition, apoptosis induction, and neovascularization blockage. Therefore, our study does not endorse the view that the sun-tanning agent MTII increases the risk of melanoma [30]. Although the detailed mechanism on PTEN stability and post-translational modification (such phosphorylation, ubiquitination etc.) remains to be elucidated, we herewith propose the working model of MTII for melanoma suppression as follows (Figure 6). After binding to MC1R, MTII elicits PTEN reactivation and AKT repression, thereby inhibiting the downstream NFκB/COX-2/PGE2 signaling to result in melanoma suppression.

The present study demonstrates the efficacy of MTII in suppressing the tumorigenicity of B16-F10 melanoma cells in vitro and in vivo. This finding is in agreement with previous studies using POMC gene therapy or α-MSH [25,32,33,34]. Furthermore, animals receiving topical MTII therapy showed no obvious changes in activity, food uptake, and body weight (data not shown), implicating topical MTII therapy seemed to be well tolerated in mice bearing melanoma. These results strongly advocate the potential of topical MTII therapy for melanoma control. However, there are still some limitations to this study. First, one single MTII dose (daily 0.1 μg per tumor for 10 days) was employed in the animal experiment, so that neither the minimally effective concentration nor the maximally tolerated dose for topical MTII therapy awaited to be determined. Second, the topical MTII therapy was in saline-based formula which is far from ideal for drug stability, absorption, and penetration. Third, despite an excellent and popular animal model, the mice B16-10 melanoma model may not faithfully recapitulate the genetic features of human melanoma. Finally, this study did not address whether MTII affected the melanoma initiation. Future experiments are warranted to overcome the above shortcoming.

PTEN is a phosphatase that negatively regulates PI3K/AKT signaling cascades. Following PTEN mutation or loss, the constitutive activation of PI3K/AKT axis renders tumor cells more aggressive and accelerates the tumor progression [35]. The present study shows that MTII treatment enhanced the protein levels of PTEN and decreased the ratio of phosphorylated PTEN in B16-F10 melanoma cells. Additionally, the blockade of surface MC1R by antibody neutralization was sufficient to abolish the MTII-induced PTEN upregulation and inhibition of anchorage-independent growth. Such MC1R-mediated PTEN upregulation seems consistent with a previous study by Cao et al. [17], which indicated that PTEN could be recruited to MC1R and protected from ubiquitination following UVB exposure in melanocytes. Interestingly, α-MSH elicits a rise of intracellular cAMP concentration in human melanoma cells with wild type MC1R but not in melanoma cells carrying mutant MC1R [36,37]. Thus, these results strongly support our hypothesis that MC1R/cAMP signaling plays a dominant role in MTII-induced melanoma suppression. However, we could exclude the probable involvement of MC4R and MC5R, which are also present in B16-F10 melanoma cells. At present, the detailed mechanism on how MTII enhanced PTEN stability remains unclear. Further studies are necessary to elucidate whether MTII regulates the expression or activities of src/casein kinase 2 or ubiquitin ligases (such as WWP1 and WWP2), thereby modulating PTEN expression in melanoma cells [11,13,35].

Chronic inflammation is a key component in the progression of tumors [38]. Our previous study showed that the inhibition of inflammatory COX-2/PGE2 signaling contributed to the POMC-mediated melanoma suppression in vitro and in vivo [24]. This is in line with the present study which shows that MTII attenuated melanoma progression through COX-2 downregulation and PGE2 reduction at nanomolar scale in B16-F10 melanoma cells as well as in melanoma tissues. Recently, a positive correlation between COX-2 and PD-L1 expression has been delineated in primary and metastatic melanoma [39]. Furthermore, the application of COX-2 inhibitor celecoxib is capable of reducing the PD-L1 protein levels in human melanoma cell lines. Therefore, the COX-2-modulating function of MTII implicates its potential to serve as an adjuvant in combination with anti-PDL1 immune checkpoint therapy for the control of advanced melanoma.

In summary, MTII exhibits anti-neoplastic effects against melanoma in vitro and in vivo. The anti-neoplastic mechanism of MTII is delineated through the activation of MC1R/PTEN signaling and the subsequent inhibition of the oncogenic AKT/NFκB/COX-2 axis. Hence, MTII may serve as a novel therapeutic adjuvant for the control of malignant melanoma.
